# Copper Acts Synergistically With Fluconazole in *Candida glabrata* by Compromising Drug Efflux, Sterol Metabolism, and Zinc Homeostasis

**DOI:** 10.3389/fmicb.2022.920574

**Published:** 2022-06-14

**Authors:** Ana Gaspar-Cordeiro, Catarina Amaral, Vânia Pobre, Wilson Antunes, Ana Petronilho, Paulo Paixão, António P. Matos, Catarina Pimentel

**Affiliations:** ^1^Instituto de Tecnologia Química e Biológica António Xavier, Universidade Nova de Lisboa, Oeiras, Portugal; ^2^Centro de Investigação da Academia Militar (CINAMIL), Unidade Militar Laboratorial de Defesa Biológica e Química (UMLDBQ), Lisbon, Portugal; ^3^Unidade de Infeção, Faculdade de Ciências Médicas, Chronic Diseases Research Centre – CEDOC, NOVA Medical School, Universidade NOVA de Lisboa, Lisbon, Portugal; ^4^Laboratório de Patologia Clínica – SYNLAB, Hospital da Luz, Lisbon, Portugal; ^5^Egas Moniz Interdisciplinary Research Centre, Egas Moniz Higher Education Cooperative, Caparica, Portugal

**Keywords:** copper, yeast, antifungal, stress response, Candida, *zap1*, zinc

## Abstract

The synergistic combinations of drugs are promising strategies to boost the effectiveness of current antifungals and thus prevent the emergence of resistance. In this work, we show that copper and the antifungal fluconazole act synergistically against *Candida glabrata*, an opportunistic pathogenic yeast intrinsically tolerant to fluconazole. Analyses of the transcriptomic profile of *C. glabrata* after the combination of copper and fluconazole showed that the expression of the multidrug transporter gene *CDR1* was decreased, suggesting that fluconazole efflux could be affected. In agreement, we observed that copper inhibits the transactivation of Pdr1, the transcription regulator of multidrug transporters and leads to the intracellular accumulation of fluconazole. Copper also decreases the transcriptional induction of ergosterol biosynthesis (*ERG*) genes by fluconazole, which culminates in the accumulation of toxic sterols. Co-treatment of cells with copper and fluconazole should affect the function of proteins located in the plasma membrane, as several ultrastructural alterations, including irregular cell wall and plasma membrane and loss of cell wall integrity, were observed. Finally, we show that the combination of copper and fluconazole downregulates the expression of the gene encoding the zinc-responsive transcription regulator Zap1, which possibly, together with the membrane transporters malfunction, generates zinc depletion. Supplementation with zinc reverts the toxic effect of combining copper with fluconazole, underscoring the importance of this metal in the observed synergistic effect. Overall, this work, while unveiling the molecular basis that supports the use of copper to enhance the effectiveness of fluconazole, paves the way for the development of new metal-based antifungal strategies.

## Introduction

Invasive fungal infections (IFIs) are a major health concern, affecting many people worldwide and causing more than 1.2 million deaths per year (Bongomin et al., 2017). IFIs caused by the yeast *Candida*, also named invasive candidiasis, represent the most common fungal disease among hospitalized patients receiving immunosuppressive or intensive antibacterial therapies (Pfaller and Diekema, 2007; Paiva et al., 2016). *Candida* blood stream infections, known as candidemias, are the prevalent form of invasive candidiasis and a serious problem in intensive care units, where between half to two-thirds of these episodes occur (Tabah et al., 2012; Paiva et al., 2016). *Candida albicans* is the predominant causative organism of candidemia, but recent data have shown a marked epidemiologic shift toward non-albicans species, such as *Candida glabrata* and *Candida parapsilosis*, which are more resistant to antifungals (Pfaller et al., 2014).

Current invasive candidiasis treatment guidelines include the antifungal fluconazole as a primary therapeutic option (Pappas et al., 2016). Fluconazole acts by inhibiting lanosterol 14-α-demethylase (Erg11), a heme-containing cytochrome P450 enzyme, involved in the biosynthesis of ergosterol, an important component of fungal membranes (Berkow and Lockhart, 2017). Inhibition of Erg11 leads to the accumulation of toxic methylated sterols in the membrane, which arrests cell growth and division (Watson et al., 1989; Kelly et al., 1995; Berkow and Lockhart, 2017). The fungistatic rather than the fungicidal properties of fluconazole together with its intensive use and misuse have paved the way for the appearance of resistant clinical isolates, with *C. glabrata* standing out among them (Pfaller et al., 2014). In fact, invasive *C. glabrata* infections are associated with high rates of morbidity and mortality plausibly because of the emergence of resistance to available antifungals (Whaley and Rogers, 2016; Pfaller et al., 2019). Also the ability of the yeast to escape the immune system and thrive in the hostile host environment is acknowledged as contributing to that scenario (reviewed in Kasper et al., 2015). In *C. glabrata*, the development of azole resistance has been almost exclusively associated with the presence of activating mutations in the Zn_2_Cys_6_ transcription factor Pdr1 (Tsai et al., 2006; Vermitsky et al., 2006). These mutations lead to the overexpression of genes encoding drug efflux proteins (Cdr1 and Cdr2), which results in the decrease of fluconazole within cells (Sanglard et al., 1999; Tsai et al., 2006; Berkow and Lockhart, 2017).

The antimicrobial properties of copper have been known for centuries and stem from its toxicity, when present above physiological needs (Smith et al., 2017). Copper also plays a central role at the host–pathogen axis. While from the host side, it is required for the differentiation and maturation of immune cells and is used as a weapon by phagocytes, as part of the nutritional immune response (García-Santamarina and Thiele, 2015), from the pathogen perspective, a fine-tuned copper homeostatic machinery is critical for survival within the host (Mackie et al., 2016).

Inspired by the antifungal properties of copper, several authors have combined this metal with fluconazole and showed that this could be a promising strategy, since the combination resulted in greater antifungal activity (Ząbek et al., 2015; Hunsaker and Franz, 2019b; Hunsaker et al., 2020). However, the mechanism underlying such synergism has yet to be elucidated.

In this study, using a genetic and biochemical approach, we investigated how copper and fluconazole act synergistically against *C. glabrata*. Our data indicate that an excess of copper inhibits the activity of Pdr1, which leads to the accumulation of fluconazole and causes abnormal sterol biosynthesis. In addition, we identified zinc homeostasis as a central player in copper fluconazole synergism, which puts zinc under the spotlight as a putative target for future antifungal investment.

## Materials and Methods

### Strains, Growth Conditions

The strains used in this study are listed in Supplementary Table 1. Yeast species were maintained in YPD agar plates. Unless otherwise stated, all assays were performed in SC medium pH 5.5 (Complete Supplement Mixture: 0.77 g/L; yeast nitrogen base w/o amino acids and ammonium sulfate 1.7 g/L; ammonium sulfate 5.4 g/L; glucose 2%). Unless otherwise stated, yeast cultures were grown until exponential phase (OD_600_ 0.8) and treated with 625 μM CuSO_4_, 32 μg/mL fluconazole, or with both compounds for 24 or 48 h. For fungicidal assays, serial dilutions of treated or untreated cultures were plated on YPD agar plates for colony forming units (CFUs) count. Whenever the CFU count was lower than that corresponding to OD_600_ 0.8 (i.e., time point 0 h), the treatment was considered fungicidal. For zinc supplementation assays 4 mM of ZnSO_4_ was used. Spot assays were carried out by spotting 5 μL of sequential dilutions (*C. glabrata*: from 8 × 10^6^ to 80 cells/mL and *Saccharomyces cerevisiae*: 4 × 10^6^ to 40 cells/mL) of exponentially growing cultures onto SC agar plates containing the indicated treatments. Unless otherwise stated, the plates were incubated for 48 h at 37°C. Spot assays were repeated at least three times. To construct the *C. glabrata* Δ*zap1* mutant (Cg Δ*zap1*), a histidine cassette was constructed by amplifying Cg*HIS3* gene with primers for the flanking region of the Cg*ZAP1* gene (pZAP1_KO_FW: 5′CATAATT GAGTATAAAAAGGACTACAAAAGCTAAGAAACTACAGAC CGGCTTGGGTACAAGAGGATAAC3′/ZAP1_KO_RV: 5′GGA CGAGTTGGAGTTATGTCTATATATTTATTGGACTATGTACA GTATAGTGAATGGGCAATGCTATGGTGTTGTTGAAACCA CCACGAG3′). *C. glabrata* competent cells and transformation were performed with the Zymo Transformation Kit (Zymo Research), according to manufacturer’s instructions. Mutant cells were confirmed by colony PCR using specific *ZAP1* primers (ZAP1_A1: 5′TGATTGTGGTGGGAGATGC3′ and ZAP1_A4: 5′GTTGATCACAGCGAGAAGC3′), followed by fragment digestion with *Xcm*I. To construct the *pZAP1* plasmid, the C*gZAP1* gene was amplified by PCR with primers for the flanking region of this gene (ZAP1_A1N: 5′AATGCAAGAGGGACAAATCG3′ and ZAP1_A4). The PCR product was inserted into the *Sma*I-digested pCgACT14 vector using T4 DNA ligase (New England Biolabs Inc.), according to manufacturer’s instructions.

### Pdr1 Transactivation Assay

The transactivation activity of *C. glabrata* Pdr1 was investigated using a *LexA*-based one-hybrid system. The *CgPDR1* ORF (CAGL0A00451g) was amplified by PCR using specific oligonucleotides (*PDR1_ATG_*II*_*FW: 5′ATGCAAACATTAGA AACTACATCAAAATC3′/PDR1_*Kpn*I_Rv: 5′ccGGTACCTCAC AAGTAAACATCAGAAAATAGGTC3′). The PCR product was digested with *Kpn*I and inserted into the *Sma*/-*Kpn*I digested Yap8-LexA plasmid (Menezes et al., 2004), in frame with the *LexA* sequence. The plasmid (*LexA-PDR1*) sequence was confirmed by sequencing. The *S. cerevisiae* EGY48 strain was then co-transformed with *LexA-PDR1* and pSH18-34 (Thermo Fisher), which contains eight *LexA* operators fused to the *LacZ* reporter gene. Positive transformants were selected in SD agar plates, after 48 h incubation at 30°C. For the transactivation assay, the co-transformed strain was grown in SD medium until exponential phase and left untreated or treated with 64 μg/mL of fluconazole, 625 μM of CuSO_4_, or with both compounds, for 2 h. Total proteins were extracted from cell cultures as described in Pimentel et al. (2012), resolved in a 10% SDS-PAGE and immunoblotted using a monoclonal anti-β-galactosidase antibody and a monoclonal anti-Pgk1 antibody. Pgk1 was used as loading control. Immunoblots were repeated twice, with different protein extracts.

### Quantitative RT-PCR

*Candida glabrata* cultures were grown under the indicated conditions, harvested by centrifugation and RNA was isolated using the phenol:chloroform method (Collart and Oliviero, 1993). RNA samples were treated with DNase and purified using the RNeasy Kit (Qiagen). Total RNA (1 μg) was reverse transcribed with “Transcriptor reverse transcriptase” (Roche), according to the manufacturer’s instructions. qPCR reactions were performed in the LightCycler 480 II Real-Time PCR System (Roche). Relative standard curves were constructed for each gene, using serial dilutions of cDNA. The relative expression of the genes was calculated by the relative quantification method with efficiency correction, using the LightCycler 480 Software 1.5 (Roche). The *RPL10* gene (CAGL0K12826g), which encodes an ortholog of the *S. cerevisiae* ribosomal L10 protein, was used as reference gene. All assays were performed using biological triplicates and technical duplicates. The following primer pairs were used: *CDR1*: 5′CATGGCCACTTTTGGTCTTT3′/5′CAGCAATGGAGACAC GCTTA3′; *PDR1*: 5′CAGCAATGGAGACACGCTTA3′/5′AG TGGGCACGTCAGAGACAG3′; *ERG11*: 5′TATGGTCGCCTTG CCATT3′/5′GACCCATGGGATCCAGTAGA3′; *ZAP1*: 5′GAAG CCTTACGAGTGCCATA3′/5′TGCACTTCAGTGGTTTCTCA3′; *RPL10*: 5′GAGATTCTTTCCACTTGAGAGTCAGA3′/5′CTC TCATACCTTGTTGCAATCTATCC3′.

### ROS Quantification

Flow cytometry analysis for ROS detection was performed as described by da Silva et al. (2018). Cultures grown for 24 h in the presence of the indicated compounds were harvested by centrifugation, washed with PBS and resuspended in PBS containing 5 μg/mL of dihydrorhodamine 123 (DHR123). After incubation in the dark at room temperature for 2 h, cells were washed with PBS and filtered. Assays were performed in an S3e cell sorter (Bio-rad) and DHR123 signal was detected using the 488 nm excitation laser and the FL1 detection channel. Unstained cells were used to establish the gate of cells exhibiting positive DHR123 signal. A total of 100,000 gated cells were counted for each sample. Five biological replicates of each condition were analyzed and the average and SD of the mean of the FL1 (DHR123 signal) peak area was calculated.

### Cell Viability Assays

Cultures grown for 24 h in the presence of the indicated compounds were harvested by centrifugation, washed with PBS and resuspended in PBS containing 50 μg/mL of propidium iodide (PI). After a 15 min incubation period, samples were washed with PBS and filtered. Assays were performed in an S3e cell sorter (Bio-rad) and PI signal was detected using the 488 nm excitation laser and the FL3 detection channel. Unstained cells were used to establish the baseline of cells exhibiting positive PI signal. Five biological replicates of each condition were analyzed and a total of 100,000 cells were counted in each sample.

### Quantification of Intracellular Metals

Cultures were grown under the indicated conditions for 24 h, harvested and washed with 10 mM EDTA and metal-free water as described in Gaspar-Cordeiro et al. (2018). Total copper and zinc cellular contents were measured by inductively coupled plasma atomic emission spectroscopy (ICP-AES) at REQUIMTE – LAQV, Universidade Nova de Lisboa, Caparica, Portugal. Data was normalized against OD_600_. All assays were made using biological quadruplicates.

### Sterol Identification and Quantification by Gas Chromatography–Mass Spectrometry

Sterol extraction and gas chromatography–mass spectrometry (GC-MS) analyses were performed according to the protocols described by Morio et al. (2012) and Demuyser et al. (2017), with minor adaptations. Briefly, cultures grown for 24 h in the presence of the indicated compounds were harvested by centrifugation. Pellets were resuspended in saponification medium (25 g of KOH, 36 mL of distilled water and made up to 100 mL with 100% ethanol), vortexed and incubated for 1 h at 80°C. After addition of 1 mL MilliQ water, 4 mL of hexane and 50 μg of cholestane (internal standard), samples were vortexed and the hexane layer recovered. Hexane was evaporated and samples were derivatized with sylliating mixture (85,432; Sigma), for 30 min. An additional 500 μL of hexane was added and samples were centrifuged at 2500 × *g* for 5 min and supernatant was recovered. For the GC-MS analysis, 1 μL of each sample was injected into a DB5 column (5% Phenyl, 95% Dimethyl Polysiloxane) in spitless mode using helium as carrier gas at a flow rate of 1 mL/min. The inlet was held at 200°C. The column oven was held at 50°C for 1 min, and then ramped up, at a rate of 50°C per min, to 260°C, followed by a ramp rate of 4°C per min up to the final temperature of 320°C, and then held for 5 min. The total run time was 25.2 min. Analyses were performed at REQUIMTE – LAQV, Universidade Nova de Lisbon, Caparica, Portugal. Sterols were identified according to their retention time (relative to cholestane) and by comparison with GC-MS profiles described in Müller et al. (2017).

### Ergosterol Quantification by HPLC

Cultures were left untreated or treated as indicated, for 24 h. Ergosterol extraction and quantification was done as described in Gaspar-Cordeiro et al. (2020), adapted from Pais et al. (2020). Analyses were performed using a Waters 2695 Alliance HPLC system (Waters Chromatography, Milford, MA, United States) equipped with a Waters 486 Absorbance Detector set at 282 nm. Samples (10 μL) were injected into a Symmetry C18 column (4.6 × 250 mm, 5 μm particle size, Waters Chromatography, Milford, MA, United States), using 95% methanol as eluent in isocratic mode at a 1.1 mL/min flow rate. Column temperature was held at 30°C and samples were kept at 10°C. The Empower 2 software (Waters Chromatography, Milford, MA, United States) was used for data acquisition. The retention time of the compound (15.7 min) was compared with standards for identification and the peak area was used for quantification. A standard curve prepared with commercial ergosterol was plotted to determine the amount of ergosterol present in each sample. All assays were performed using biological quadruplicates.

### Fluconazole Quantification by HPLC

*Candida glabrata* cultures were grown under the indicated conditions for 24 h, harvested, resuspended in 2 mL MilliQ water and normalized against OD_600_. Tinidazole (100 μg) was added to samples prior to extraction. Extraction was then carried out using glass beads and methanol. After 30 s of vortex agitation, samples were incubated at 30°C and 200 rpm for 1 h, followed by centrifugation at 4000 × *g* for 7 min at 4°C. The supernatant was then recovered and centrifuged again at 9500 × *g* for 10 min, to remove any remaining debris. Analyses were performed using a Waters 2695 Alliance HPLC system (Waters Chromatography, Milford, MA, United States) equipped with a Waters 486 Absorbance Detector set at 245 nm. Samples (80 μL) were injected into a reverse phase Symmetry C18 column (4.6 × 250 mm, 5 μm particle size, Waters Chromatography, Milford, MA, United States) using an isocratic eluent mixture of ammonium acetate buffer/acetonitrile/methanol (80:15:5% v/v) at 0.7 mL/min flow rate. Column temperature was held at 30°C and samples were kept at 10°C. The Empower 2 software (Waters Chromatography, Milford, MA, United States) was used for data acquisition. The retention time of fluconazole (13.4 min) was compared with standards for identification, and the peak area was used for quantification. Quantification of fluconazole was normalized to the internal control (tinidazole, retention time 10.4 min). Standard curves for fluconazole and tinidazole were plotted to measure the amount of compound present in each sample. All assays were performed using biological quadruplicates.

### Immunoblotting

The BVGC3 strain was grown until exponential phase (OD_600_ 0.8), treated with 625 μM CuSO_4_, 32 μg/mL fluconazole, or with both compounds for up to 2.5 h and harvested. Total proteins were extracted from cell cultures as described in Pimentel et al. (2012). Protein extracts (50 μg) were resolved in a 12% SDS/PAGE gel and immunoblotted. An anti-HA-Peroxidase high affinity rat monoclonal antibody (Roche) was used to detect the HA-tagged version of Erg11 (Vu et al., 2019). Pgk1 was used as loading control and detected using a monoclonal antibody (Life Technologies). Immunoblots were repeated at least twice with different protein extracts.

### RNA-Sequencing and Data Analysis

*Candida glabrata* ATCC2001 was grown to exponential phase (OD_600_ 0.8) in SC medium and left untreated or treated with 1.25 mM CuSO_4_, 32 μg/mL fluconazole, or a combination of both for 1 h. Biological triplicates were used for each condition. Cells were harvested and RNA was isolated. RNA samples were treated with DNase and purified using the RNeasy Kit (Qiagen). Library preparation and sequencing was done at the Genomics Facility of Instituto Gulbenkian da Ciência, Portugal, using the SMART-SEQ2 protocol, adapted from Picelli et al. (2014), and an Illumina NextSeq500 platform (single end, 75-bp read length, 20 M reads), respectively. The quality of the RNA-sequencing (RNA-Seq) raw data was analyzed using the FastQC tool. We mapped the reads against *C. glabrata* genome (GCA_000002545.2 downloaded from NCBI genome database) using Bowtie2 program and obtained more than 90% of aligned reads (Langmead and Salzberg, 2012). The mapping files were sorted by genomic position using Samtools and the quantification of the transcripts expression was done using the FeatureCounts software (Li et al., 2009; Liao et al., 2014). Differential expression analyses were done with the R package edgeR (Robinson et al., 2010). We considered all transcripts with a false discovery rate (FDR) correction of the *p*-value lower than 0.05 as significant and we further filtered our results by establishing cut-offs of expression (Log_2_CPM – counts per million – higher than 3) and fold change (FC) higher than 2. The functional annotation was performed with FungiFun (Priebe et al., 2011).

### Checkerboard Assays

The assays were performed in SC medium using 96-well flat bottom plates, according to the CLSI standard M27-A3 (CLSI M27, 2017), following the protocol described in Marchetti et al. (2000) and Fiori and Van Dijck (2012). Fluconazole and copper stock solutions were prepared in bi-distilled water, filtered and further diluted in medium. Twofold dilutions were prepared for each drug so that final concentrations ranged from 128 to 0.125 μg/mL for fluconazole and 2500 to 39 μM for CuSO_4_. A 2 × 10^6^ cells/mL suspension obtained from a single colony was diluted to 3 × 10^3^ cells/mL, in SC medium, to prepare the final inoculum. MIC endpoints were defined as the lowest drug concentration leading to a growth reduction higher than 50%, as determined by measuring OD_600_. The type of interaction for each copper and fluconazole combination tested was determined by calculating the fractional inhibitory concentration (FIC) index (∑FIC = FIC_*Cu*_ + FIC_*Fluc*_). The MIC for fluconazole (MIC_*Fluc*_) and copper (MIC_*Cu*_) were determined to be 64 μg/mL and 625 μM, respectively. For each drug combinations the FIC was calculated as follows: FIC_*Cu*_ = MIC_*Cu+Fluc*_/625 and FIC_*Fluc*_ = MIC_*Fluc+Cu*_/64. FIC index ≤ 0.5 indicates a synergistic effect.

### Scanning Electron Microscopy

*Candida glabrata* cultures were grown under the indicated conditions for 24 h and harvested by centrifugation. Samples were fixed with a mixture of 0.4% glutaraldehyde and 4% formaldehyde in sodium cacodylate buffer 0.1 M for 18 h at room temperature, followed by three washing steps with sodium cacodylate buffer 0.1 M. Samples were dehydrated by sequential washing with four ethanol solutions (50, 70, 90 and 100%). After the last dehydration step, ethanol was removed and tert-butyl alcohol was added. Samples were incubated at 30°C for 1 h, placed on ice until completely frozen, freeze-dried by lyophilization and kept at room temperature until the scanning electron microscopy (SEM) analyses were performed. Samples were gold sputtered with 8 nm gold in an electron sputter (Cressington 108) and imaged in a Hitachi SU8010 scanning electron microscope, at 1.5 kV. Approximately 120 cells were counted per condition and the proportion of cells exhibiting surface abnormalities was calculated.

### Transmission Electron Microscopy

*Candida glabrata* cultures were grown under the indicated conditions for 24 h, harvested by centrifugation and fixed in 3% glutaraldehyde in 0.1 M sodium cacodylate buffer pH 7.3. Following primary fixation for 2 h at 4°C and wash in the cacodylate buffer, cells were, pelleted and embedded in 2% agar for further processing. Samples were further fixed for 3 h in 1% osmium tetroxide in 0.1 M sodium cacodylate buffer pH 7.3. Then, samples were washed in 0.1 M acetate buffer, pH 5.0 and fixed in 1% uranyl acetate in the same buffer for 1 h. Dehydration was carried out with increasing concentrations of ethanol. After passing through propylene oxide, samples were embedded in Epon-Araldite, using SPI-Pon as an Epon 812 substitute. Thin sections were made with diamond knives and stained with 2% aqueous uranyl acetate and Reynold’s lead citrate. The stained sections were analyzed and photographed in a JEOL 1200-EX electron microscope.

### Data Availability

RNA-sequencing raw data is available at Gene Expression Omnibus (GEO) under the accession number GSE162741.

## Results

### Copper Acts Synergistically With Fluconazole

**FIGURE 1:**
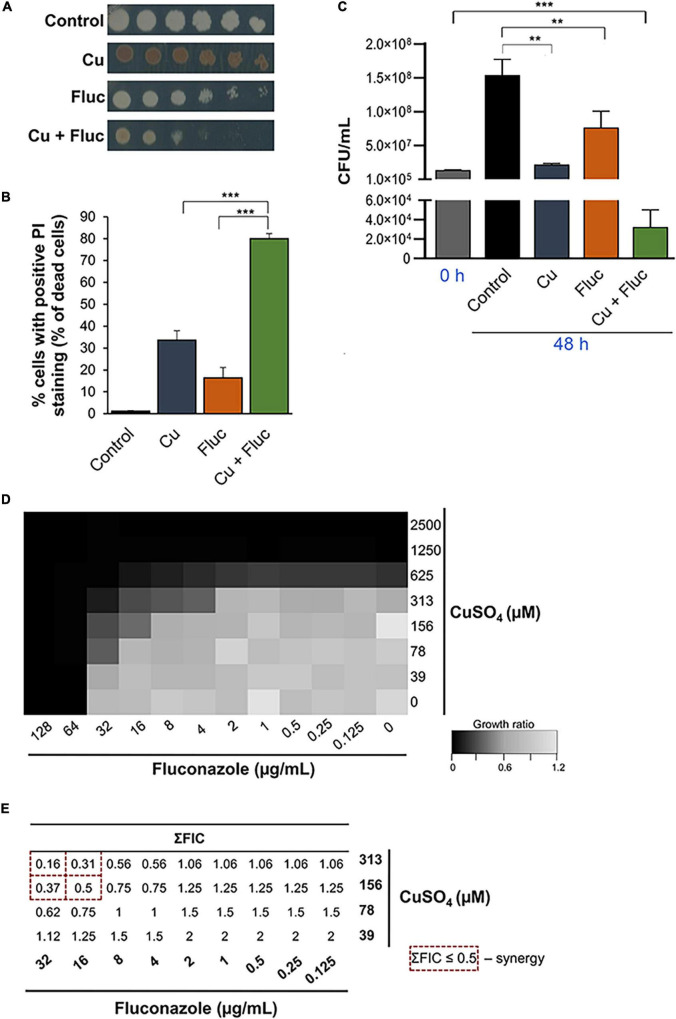
Copper acts synergistically with fluconazole. **(A)** Growth sensitivity of *Candida glabrata* in SC agar plates (Control) containing 32 μg/mL of fluconazole (Fluc), 625 μM CuSO_4_ (Cu), or a combination of both (Cu + Fluc). Growth was recorded after 48 h at 37°C. **(B)** Detection of dead cells by flow cytometry using propidium iodide staining (PI) under control conditions or after treatment with 625 μM CuSO_4_ (Cu), 32 μg/mL fluconazole (Fluc), or both (Cu + Fluc) for 24 h. **(C)** Fungicidal effect of Cu + Fluc treatment. Cells treated as described in **(B)** were grown for 48 h and plated on YPD agar plates for CFU count. Significance of differences was calculated using Student’s *T*-test (**p-value < 0.05; ***p-value < 0.005). **(D)** Checkerboard assays. Cells were exposed to all the possible combinations of CuSO_4_ and fluconazole (ranging between 2500 and 39 μM for CuSO_4_ and 128 and 0.125 μg/mL for fluconazole). Growth ratios were evaluated after 24 h at 37°C by measuring OD_600_ and normalizing it relative to untreated controls (see gradient bar). **(E)** Fractional inhibitory concentration (FIC) index (∑FIC) was calculated for the indicated combinations of fluconazole and CuSO_4_. The dashed red squares indicated the Cu + Fluc concentrations where synergy is observed.

Growing evidence indicates that copper potentiates the antifungal activity of fluconazole against opportunistic pathogenic yeasts (Ząbek et al., 2015; Hunsaker and Franz, 2019b; Hunsaker et al., 2020). Here, we confirmed that the combination of copper and fluconazole impairs the growth of *C. glabrata* at concentrations where these compounds cause only mild growth defects when used separately (Figure 1A). Accordingly, by flow cytometry using PI, a membrane impermeable dye generally excluded from viable cells, we found that, when used together, copper and fluconazole strongly increased PI staining by more than twofold compared with individual treatments (Figure 1B). Noteworthy, clinical isolates and other laboratory strains exhibited a similar growth phenotype (Supplementary Figures 1A,B), indicating that the effect is not restricted to the most used strain in this study (ATCC2001). Most importantly, we found that when copper and fluconazole are put together they have fungicidal rather than fungistatic activity as they do when they are added to cultures separately (Figure 1C). Consistent with previous reports (Lachke et al., 2000), we observed that *C. glabrata* colonies exhibited a brown pigmentation in the presence of copper, presumably as a result of CuS mineralization on the cell surface (Yu et al., 1996). Notably, co-treatment with fluconazole decreased this pigmentation (Figure 1A).

The interaction between copper and fluconazole was further investigated using checkerboard microdilution assays. We tested the effect on cell growth of all possible combinations of copper and fluconazole, ranging from 2500 to 39 μM and 128 to 0.125 μg/mL, respectively. The results are summarized in Figures 1D,E. The MIC_*Fluc*_ was 64 μg/mL and MIC_*Cu*_ 625 μM. Copper reduced the MIC_*Fluc*_ 4-fold (16 μg/mL) and 16-fold (4 μg/mL) when added at concentrations of 156 and 313 μM, respectively. Fluconazole decreased the MIC_*Cu*_ fourfold (156 μM) and eightfold (78 μM) when combined with copper at concentrations of 16 and 32 μg/mL, respectively (Figure 1D). Synergistic effects (ΣFIC ≤ 0.5) between copper and fluconazole were observed for concentrations equal to or higher than 156 μM (CuSO_4_) and 16 μg/mL (fluconazole), as depicted in Figure 1E. The effect was also observed in a *C. glabrata* strain with a lower MIC for fluconazole (CgΔ*cdr1*, Supplementary Figures 1C,D).

### Differences in Copper Accumulation Do Not Explain the Synergistic Effect Between Copper and Fluconazole

**FIGURE 2:**
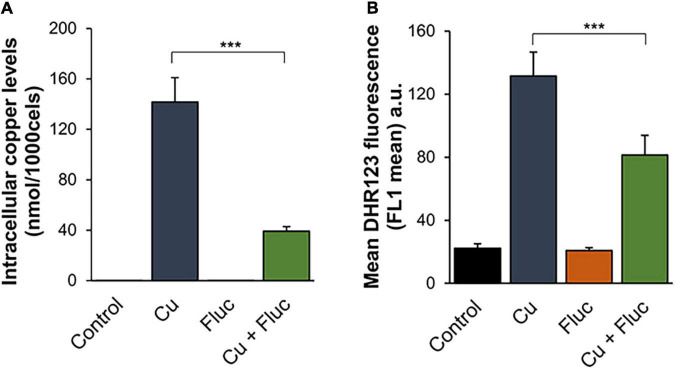
Differences in copper accumulation do not explain the synergistic effect between copper and fluconazole. **(A)** The copper content of *Candida glabrata* cells left untreated (Control) or treated with 625 μM CuSO_4_ (Cu), 32 μg/mL fluconazole (Fluc), or both (Cu + Fluc) for 24 h was determined by ICP-AES. **(B)** ROS detection in *Candida glabrata* cells left untreated (Control) or treated 625 μM CuSO_4_ (Cu), 32 μg/mL fluconazole (Fluc), or both (Cu + Fluc) for 24 h, was performed by flow cytometry using DHR123. Values are the mean of five independent biological replicates with 100,000 cells counted per each condition. Significance of differences was calculated using the Student’s *T*-test (***p-value < 0.005).

Fluconazole is capable of forming complexes with metals, which likely increases its antifungal activity (Ząbek et al., 2015). Therefore, we first hypothesized that the observed synergism between the drug and copper (Figure 1) could be a result of the formation of a fluconazole-copper complex. However, we were unable to observe such a complex under our experimental conditions (data not shown). Similar results were reported by Hunsaker and Franz (2019b), clearly showing that a more intricate relationship should underpin the observed synergistic effect. Interestingly, those authors also demonstrated that adaptation to fluconazole in *C. albicans* required the modulation of metal homeostasis and that cells exposed to the drug accumulated more copper (Hunsaker and Franz, 2019a). In this sense, we started by testing if the synergy between copper and fluconazole in *C. glabrata* could also be due to the toxic accumulation of copper. To do so, we quantified the levels of copper in cells treated for 24 h with 32 μg/mL fluconazole, 625 μM CuSO_4_, or a combination of both compounds (hereafter referred to as Cu + Fluc). Unlike *C. albicans* (Hunsaker and Franz, 2019a), Cu + Fluc-treated *C. glabrata* cells were found to accumulate far less copper when compared with those treated with the metal alone (Figure 2A). In agreement with this observation, cells exposed to Cu + Fluc also had lower levels of ROS, a well-known mechanism of copper toxicity (Smith et al., 2017), as measured by flow cytometry using DHR123 (Figure 2B).

These data indicate that in *C. glabrata*, the antifungal synergy between copper and fluconazole cannot be attributed to exacerbated copper accumulation and that other mechanism(s), potentially specific to this organism, should underlie such effect.

### Transcriptional Response of *Candida glabrata* to Copper and Fluconazole

In yeast species, the response to copper overload requires the transcriptional activation of copper detoxification genes, such as those encoding metallothioneins (Mehra et al., 1992), by a copper-responsive transcription factor [Amt1 in *C. glabrata* (Zhou and Thiele, 1991)]. The adaptation to fluconazole also relies on the transcriptional activator, Pdr1 (Vermitsky and Edlind, 2004; Tsai et al., 2006; Vermitsky et al., 2006), which is responsible for activating multidrug efflux genes, such as *CDR1* and C*DR2* (Sanglard et al., 1999; Vermitsky and Edlind, 2004). Therefore, as a first approach to understanding the antifungal synergism between copper and fluconazole, we decided to investigate the alterations in the transcriptional landscape of *C. glabrata* exposed to Cu + Fluc and compared them with those of cells treated with fluconazole and copper separately. Differential gene expression analyses (relative to untreated cells) were conducted using edgeR (Robinson et al., 2010), and a set of restrictive parameters was applied (log_2_FC > 1, log_2_CPM > 3, and FDR < 0.05) to identify the most relevant affected pathways.

**FIGURE 3:**
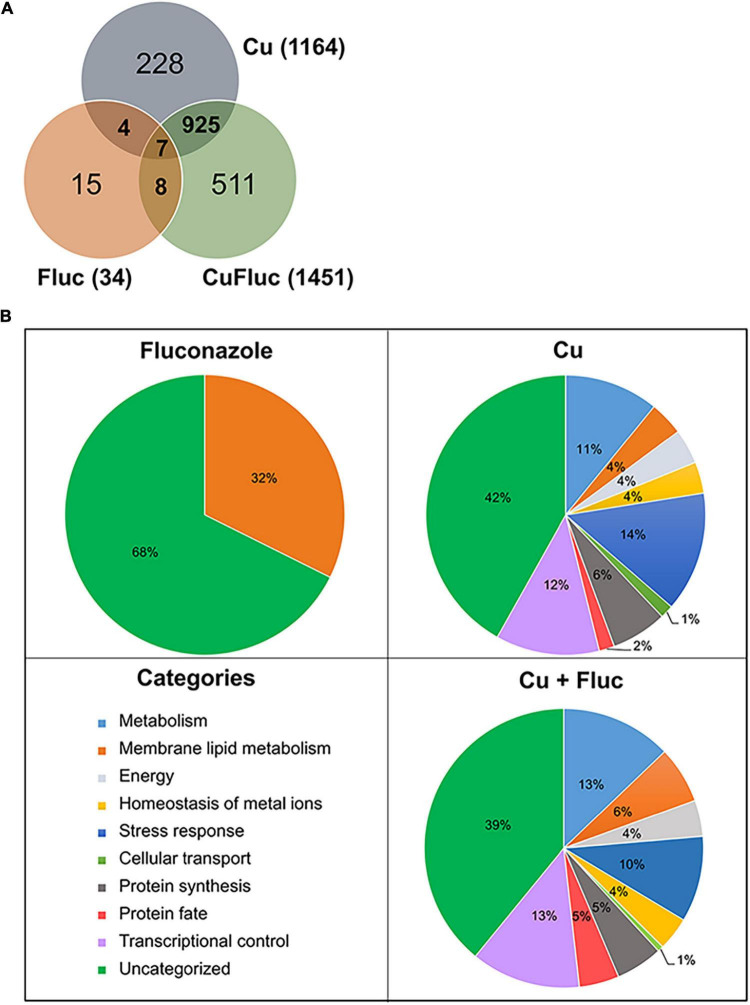
Transcriptional response of *Candida glabrata* to copper and fluconazole. **(A)** Venn diagram representing the number of genes differentially expressed. The total number of differentially expressed genes in each condition (treated versus untreated) is indicated in parentheses. **(B)** Functional categorization of the differentially expressed genes based on corresponding encoded (or putatively encoded) proteins. Only enriched categories (*p*-value < 0.05) are represented.

After exposure to fluconazole, only 34 genes were found differentially expressed, whereas copper and Cu + Fluc altered the expression of 1164 and 1451 genes, respectively. The overlapping and specific alterations of each transcriptomic profile are illustrated in Figure 3A and the genes whose expression was affected by the individual or combined treatments are listed in Supplementary Tables 2–4.

The functional categorization of the differentially expressed genes is represented in Figure 3B. Genes with altered expression under fluconazole treatment were grouped into the single category “membrane lipid metabolism.” This category includes genes of the *ERG* pathway, such as *ERG11*, *ERG1*, *ERG2*, *CYB5*, and *NCP1*, which were upregulated (Supplementary Table 3), as expected and reported by other authors (Henry et al., 2000; Whaley et al., 2014).

**FIGURE 4:**
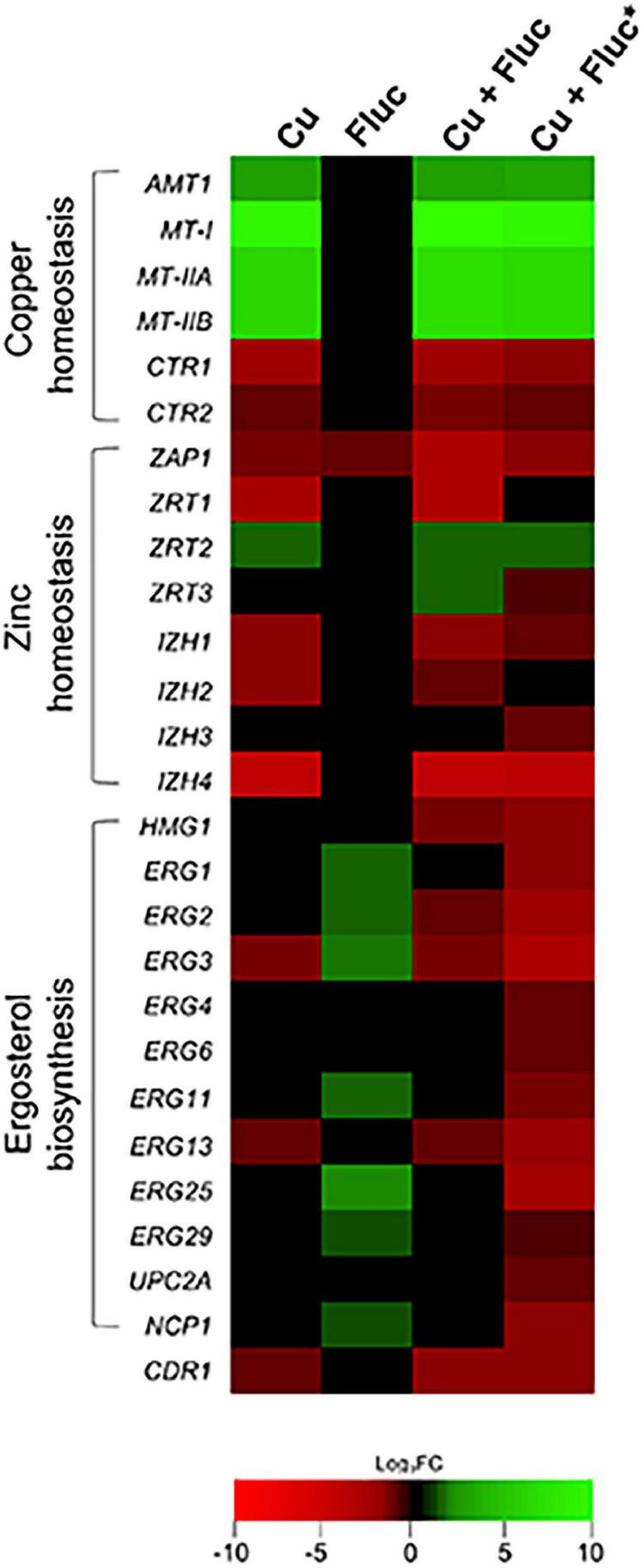
Heatmap depicting *Candida glabrata* genes belonging to the subcategories ergosterol biosynthesis, copper and zinc homeostasis, which were differentially expressed under Cu, Fluc, or Cu + Fluc conditions. For each condition, the log_2_ fold change (FC) of the selected transcripts is indicated using a color code (see the color bar). Genes significantly up- (log_2_FC > 1) or downregulated (log_2_FC < –1) in response to Cu, Fluc, or Cu + Fluc (treated versus untreated conditions) are shaded in green or red, respectively. Genes that were not differentially expressed between the treated and untreated conditions appear in black (–1 < log_2_FC < 1). The colors depicted in the Cu + Fluc* column indicate genes differentially expressed in Cu + Fluc conditions compared to Fluc conditions alone. *S. cerevisiae* gene names were used whenever the corresponding *C. glabrata* homolog name was not assigned.

The genes differentially expressed after copper and Cu + Fluc treatment fall into nine categories (Figure 3B). Both treatments showed significant enrichment in broad processes like metabolism, stress response, transcriptional control, and protein synthesis, consistent with a strong genetic reprogramming that cells should undergo. Included in the “homeostasis of metal ions” category are (1) genes involved in the copper-detoxification response, such as those encoding the metallothioneins (*MT-I*, *MT-IIA*, *MT-IIB*) and the transcriptional regulator of copper detoxification, *AMT1* (Zhou and Thiele, 1991; Mehra et al., 1992), and (2) genes involved in copper transport and compartmentalization, such as *CTR1* and *CTR2*, which are homologs of the *S. cerevisiae* genes coding for the high-affinity copper importer and the vacuolar low-affinity copper transporter, respectively (Dancis et al., 1994; Rees et al., 2004). As anticipated, the first set of genes was upregulated, while the second was downregulated (Figure 4). Under this category, we also find genes involved in Fe-S cluster biogenesis (*ISU1*, *ISA1*, *ISA2*, *CIA1*, *CIA2*, *NPB35*). Fe-S clusters are primary targets of copper toxicity (Macomber and Imlay, 2009) and their involvement in fluconazole tolerance has recently been demonstrated (Demuyser et al., 2017). Another group of genes comprised in this category code for proteins whose orthologs play a role in zinc homeostasis (Zhao et al., 1998). These genes, which include *ZAP1*, the regulator of zinc homeostasis, and some of its putative target genes, were downregulated in copper and, more prominently, in Cu + Fluc conditions (Figure 4), suggesting that zinc homeostasis might be especially affected by the latter treatment. Remarkably, *ZAP1* was also downregulated in cells exposed to fluconazole alone (Figure 4).

The “membrane lipid metabolism” category was enriched under copper and Cu + Fluc treatment. Interestingly, we noticed that several genes of the ERG pathway were repressed in response to copper and Cu + Fluc (Figure 4). This observation was even more striking when we compared gene expression under Cu + Fluc with that of cells only treated with fluconazole (Figure 4 and Supplementary Table 5). Clearly, the combination of copper and fluconazole led to the transcriptional repression of many genes relevant for ergosterol biosynthesis, among which were *UPC2A* and *ERG11* that encode the transcriptional regulator of the pathway (Whaley et al., 2014) and the target of fluconazole (Kelly et al., 1995), respectively.

The “stress response” category comprises several genes involved in oxidative stress detoxification (*CTA1*, *TRX2*, *GLR1*, *GSH2*, *TRR1*), found induced in response to both copper and Cu + Fluc (Supplementary Tables 2, 4), possibly to counteract the oxidative damage imposed by the metal (Figure 2B). The gene encoding the ATP binding cassette (ABC) transporter Cdr1, which is linked with azole resistance in *C. glabrata* (Sanglard et al., 1999), also appears under this category. Surprisingly, its expression was downregulated both by copper (log_2_FC_*Cu*_ −1.38) and Cu + Fluc (log_2_FC_*Cu+Fluc*_ −1.74), contrasting with its upregulation following treatment with fluconazole (Sanglard et al., 1999).

In summary, this analysis pinpoints two pathways known to be relevant for fluconazole adaptation that, according to the transcriptional data, might be negatively affected by the presence of copper or even worsen under Cu + Fluc conditions, namely (1) drug detoxification through Cdr1 and (2) ergosterol biosynthesis. In addition, zinc homeostasis may be deregulated when copper and fluconazole are combined, which might pose a threat because zinc is an essential metal whose intracellular levels need to be delicately balanced.

### Copper Excess Interferes With Pdr1 Activity and Leads to the Accumulation of Fluconazole

**FIGURE 5:**
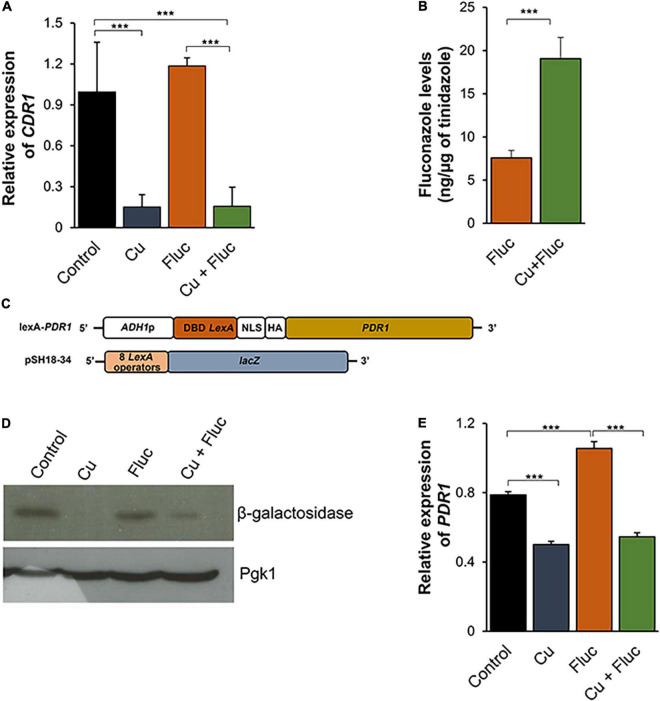
Copper treatment inhibits Pdr1 activity, downregulates *CDR1* expression and impairs fluconazole detoxification. **(A)** The expression of the *CDR1* gene in cells untreated (Control) or treated with 1.25 mM CuSO_4_ (Cu), 32 μg/mL fluconazole (Fluc), or both (Cu + Fluc), was analyzed by qRT-PCR. **(B)** The accumulation of fluconazole in cells untreated (Control) or treated with 625 μM of CuSO_4_ (Cu), 32 μg/mL fluconazole (Fluc), or both (Cu + Fluc) was measured by HPLC. The amount of fluconazole was normalized relatively to tinidazole (internal standard). **(C)** Scheme depicting the constructs used in the transactivation assay **(D)**. **(D)** *S. cerevisiae* EGY48 strain transformed with *LexA-PDR1* and pSH18-34 was grown until mid-log phase in selective media and treated (or left untreated) with 625 μM CuSO_4_ (Cu), 64 μg/mL fluconazole (Fluc), or both (Cu + Fluc) for 2 h. β-Galactosidase protein levels were analyzed by Western blot. Pgk1 was used as loading control. **(E)** The expression of the *PDR1* gene was assessed by qRT-PCR. Significance of differences was calculated using Student’s *T*-test (***p-value < 0.005).

Confirming the RNA-Seq data, qRT-PCR analysis indicated that the transcript levels of *CDR1* were noticeably downregulated in response to copper and Cu + Fluc (Figure 5A). A similar expression pattern was found for concentrations where the minimal synergistic effect (156 μM copper and 16 μg/mL fluconazole, FIC = 0.5, Figure 1D) was observed (Supplementary Figure 2). We next tested whether the repression of *CDR1* expression by copper affected the accumulation of fluconazole. For that, we quantified the intracellular levels of fluconazole by HPLC. Consistent with *CDR1* repression, we observed that cells co-treated with copper accumulate higher levels of fluconazole (Figure 5B).

In *C. glabrata*, the Zn_2_Cys_6_ transcription factor Pdr1 regulates azole resistance through the induction of multidrug efflux genes, among which is *CDR1* (Vermitsky and Edlind, 2004; Vermitsky et al., 2006). Given the ability of copper to cause mismetallation and interfere with the activity of zinc-finger transcription factors (Predki and Sarkar, 1994), we decided to test whether the metal could be affecting the transactivation activity of Pdr1 under the conditions tested. To this end, we first generated a construct containing the *C. glabrata PDR1* ORF fused to the *LexA* ORF regulated by the *ADH* promoter (*LexA-PDR1*). The *S. cerevisiae* EGY48 strain was then co-transformed with *LexA-PDR1* and the pSH18-34 plasmid, which contains the *LacZ* reporter gene, encoding β-galactosidase under the control of 8 *LexA* operators (Figure 5C). The expression of the reporter gene, which reflects the ability of CgPdr1 to recruit the basal transcription machinery, was assessed by Western blotting (Figure 5D). The results showed that copper, alone or combined with fluconazole, decreased the levels of β-galactosidase, suggesting that excessive levels of the metal (Figure 2A) negatively affect the activity of Pdr1.

As the activity of Pdr1 is controlled by several factors, including transcriptional autoregulation (recently reviewed in Moye-Rowley, 2019), we decided to measure the expression of *PDR1* by qRT-PCR under copper, fluconazole, Cu + Fluc and control conditions. Although *PDR1* was not considered differentially expressed in our RNA-Seq analysis, by qRT-PCR, we observed that, like *CDR1* (Figure 5A), its expression was partly inhibited after treatment with copper, even in the presence of fluconazole (Figure 5E). Further reinforcing these findings, using PathoYeastract (Monteiro et al., 2019) we found that 21% of the 584 genes downregulated by copper (RNA seq analysis, Supplementary Table 2) had been documented to be regulated by Pdr1 and that 52% of the remaining genes have PDRE binding motifs within their promoters and may therefore also be regulated by Pdr1.

Altogether, these results demonstrate that copper represses the expression of *CDR1*, which should explain the accumulation of the drug when copper and fluconazole are combined. The fact that the transactivation activity of Pdr1 is decreased under copper conditions further supports the idea that *CDR1* repression and fluconazole accumulation can be a consequence of Pdr1 inhibition imposed by excessive levels of the metal.

### The Combination of Copper and Fluconazole Affects Ergosterol Metabolism

**FIGURE 6:**
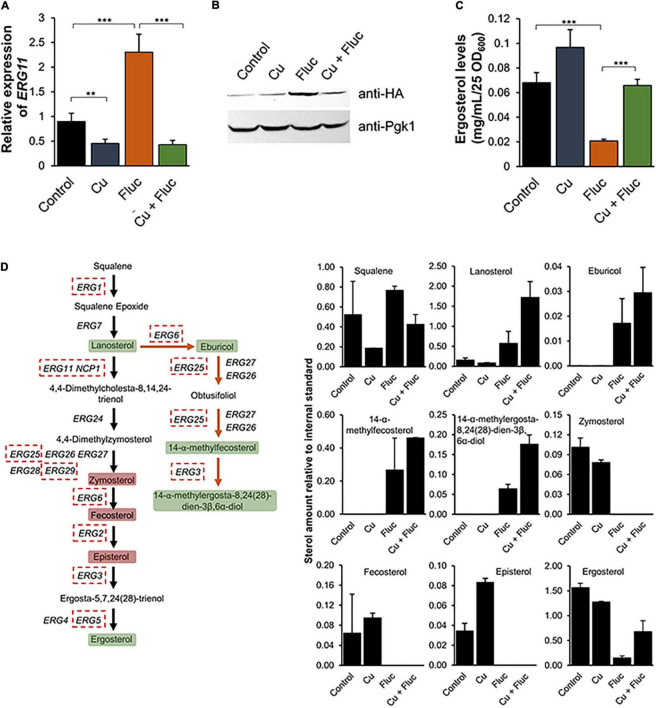
The combination of copper and fluconazole affects ergosterol metabolism. **(A)** The expression of the *ERG11* gene was determined by qRT-PCR in *Candida glabrata* cells untreated (Control) or treated with 1.25 mM CuSO_4_ (Cu), 32 μg/mL fluconazole (Fluc), or with the two compounds (Cu + Fluc). **(B)** Western blot analyses of a HA-tagged version of Erg11, after treatment with 625 μM CuSO_4_, 32 μg/mL fluconazole, or both for 2 h 30 m. **(C)** The ergosterol content of cells left untreated (Control) or treated with 625 μM of CuSO_4_, 32 μg/mL fluconazole (Fluc), or Cu + Fluc was measured by HPLC. Significance of differences was calculated using Student’s *T*-test (**p-value < 0.05; ***p-value < 0.005). **(D)** Schematic representation of the effect of Cu + Fluc on the biosynthesis of ergosterol. Dashed red boxes indicate the genes downregulated under Cu + Fluc treatment compared to fluconazole treatment, according to RNA-Seq analyses. Green boxes indicate sterol metabolites, which are more abundant after Cu + Fluc treatment, as compared to cells treated with fluconazole alone. Red boxes indicate sterol metabolites undetected by GC-MS under Cu + Fluc and Fluc conditions. The graphs indicate the relative amount of sterol intermediates in cells left untreated (Control) or treated with 625 μM of CuSO_4_ (Cu), 32 μg/mL fluconazole (Fluc), Cu + Fluc, as determined by GC-MS. Cholestane was used as an internal standard.

In response to ergosterol depletion caused by azoles, yeast cells increase the expression of the genes involved in the *ERG* pathway (*ERG* genes) (Henry et al., 2000; Whaley et al., 2014). Interestingly, our data shows that the combination of copper and fluconazole prevents this upregulation (Figure 4). Accordingly, we found that *ERG11* mRNA levels were slightly but significantly downregulated in the presence of copper, and the combination of the metal with fluconazole precluded the upregulation of *ERG11*, observed when fluconazole was added to the cultures (Figure 6A). A similar gene expression profile was observed for the minimal concentrations sufficient to trigger the synergistic effect (Supplementary Figure 2). Corroborating the transcriptional analyses, we found that Erg11 protein levels were compromised after co-treatment compared to fluconazole treatment alone (Figure 6B). Contrary to our expectations, however, Cu + Fluc-treated cells possessed higher ergosterol levels than cells treated exclusively with fluconazole, as measured by HPLC (Figure 6C).

To have a comprehensive understanding of the impact of copper and fluconazole on ergosterol metabolism, we analyzed the sterol profile of *C. glabrata* exposed to each condition, by GC-MS. A scheme summarizing the GC-MS results integrated with the transcriptomic data can be found in Figure 6D. Lanosterol, the substrate of Erg11, as well as the intermediates of the ergosterol alternative pathway [eburicol, 14-α-methylfecosterol and 14-methylergosta-8,24(28)-dien-3β,6α-diol], which are known to compromise the function of the plasma membrane (Watson et al., 1989; Kelly et al., 1995), were not detected under copper and control conditions, but accumulated when cells were treated with fluconazole. The levels of these intermediates were overall higher under the conditions of Cu + Fluc than fluconazole alone (Figure 6D), which is in line with the greater accumulation of fluconazole in the former condition (Figure 5B). Zymosterol and fecosterol (late-stage intermediates downstream of lanosterol) could not be detected in any of the fluconazole conditions (Figure 6D). Corroborating the HPLC analysis (Figure 6C), by GC-MS, we confirmed that copper alleviates the ergosterol depletion caused by fluconazole (Figure 6D).

**FIGURE 7:**
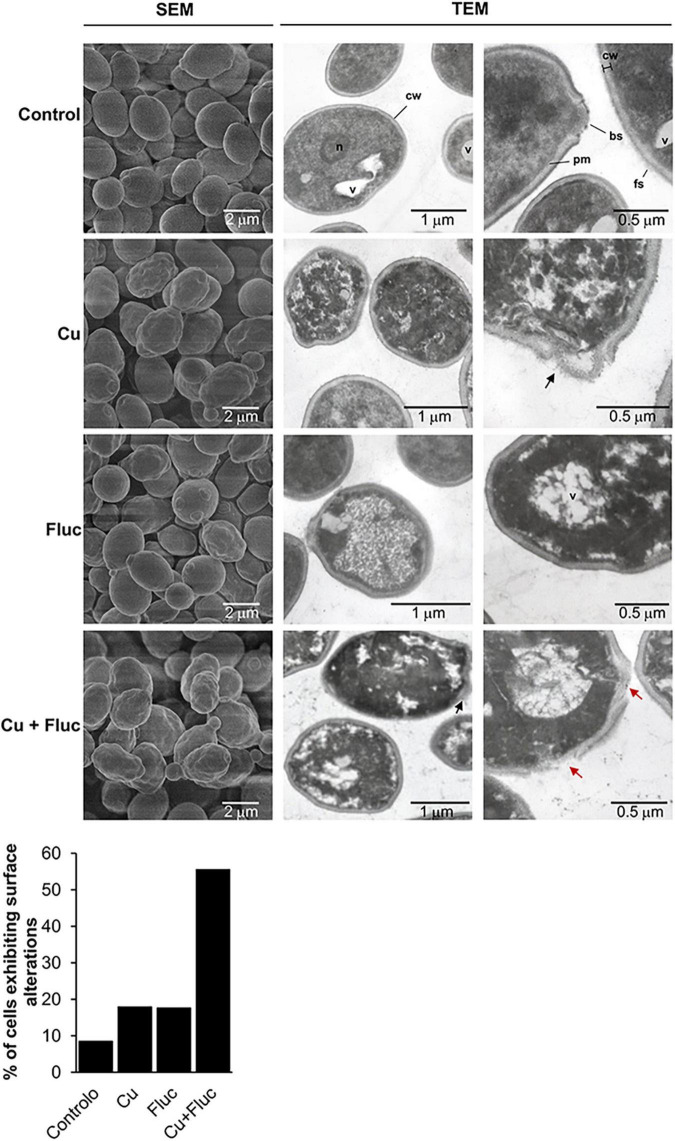
Scanning electron microscopy (SEM) and transmission electron microscopy (TEM) images of *Candida glabrata* cells left untreated (Control), or treated with 625 μM CuSO_4_ (Cu), 32 μg/mL fluconazole (Fluc), or both (Cu + Fluc) for 24 h. Control cells have regular cell walls (cw), from which fibrillar structures (f) protrude, well defined and continuous plasma membrane (pm) and visible vacuoles (v), nucleus (n), and bud scars (bs). Cells treated with Cu, Fluc or Cu + Fluc exhibit irregular surfaces and disorganized cytoplasm. Cu and Cu + Fluc stresses also induce defects in the cell wall (black arrows) and in the latter condition, loss of cell wall integrity (red arrows) was observable in some cells. The graph indicates the percentage of cells exhibiting surface abnormalities. A total of 120 cells per condition were imaged by SEM and those with irregular surfaces were counted.

As alterations in the sterol content of the yeast cell can affect membrane proteins that build and shape the cell wall and extracellular matrices (Kodedová and Sychrová, 2015), we used SEM to evaluate the impact of copper and fluconazole on cell surface morphology. *C. glabrata* untreated cells have a well-defined oval shape with a smooth surface (Figure 7), whereas copper- and fluconazole-treated cells sporadically exhibited a rough and irregular surface. In contrast, more than 50% of the cells treated with copper and fluconazole simultaneously have pronounced surface irregularities (Figure 7). The morphology of untreated and treated cells was also observed using transmission electron microscopy (TEM) (Figure 7). Untreated cells have normal cell walls (cw) with a thin electron-dense outer layer with protruding fibrillar structures (fs). A continuous inner plasma membrane (pm) delimits an electron-dense and conserved cytoplasm in which nucleus (n) and vacuoles (v) are visible. Treatment with copper and fluconazole induced structural changes at the periphery and inside the cells, which were more pronounced when both treatments were combined. Among the ultrastructural alterations were: irregular cell wall and plasma membrane (all treatments), decreased cytoplasmic density and disorganization of the cytoplasm (all treatments), clear defects in the cell wall (Cu and Cu + Fluc treatments, black arrows in Figure 7) and, in some cells, loss of cell wall integrity (Cu + Fluc treatment, red arrows in Figure 7). Both the accumulation of copper (Figure 2A) and the alteration of sterol metabolism, despite the higher levels of ergosterol (Figure 6D), should certainly contribute to the Cu + Fluc phenotype.

### Co-treatment of *Candida glabrata* Cells With Copper and Fluconazole Causes Zinc Depletion

The striking observation that several genes presumably involved in zinc sensing and transport were downregulated after fluconazole, copper and Cu + Fluc treatment (Figure 4) led us to investigate whether zinc homeostasis could be affected. Included in this set of genes was *ZAP1* that, in yeasts, encodes a zinc-finger transcription factor that acts as master regulator of zinc homeostasis (Eide, 2009).

**FIGURE 8:**
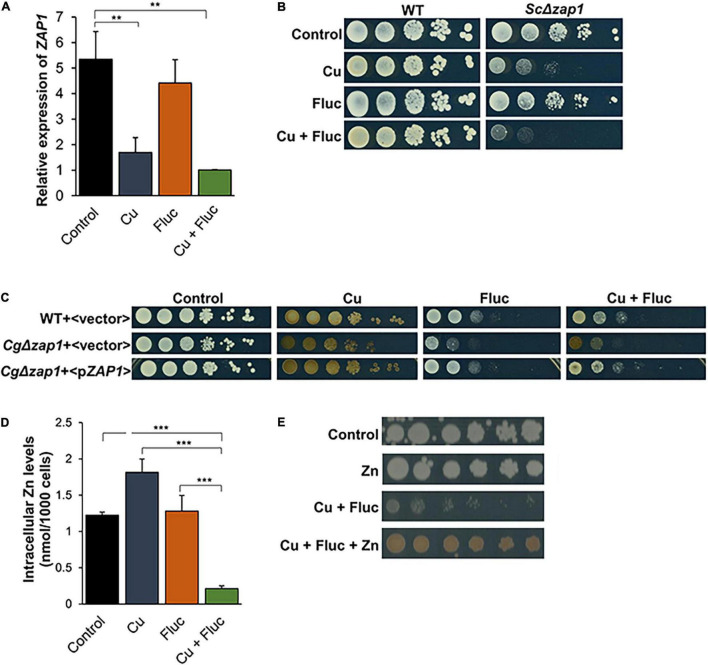
Zinc homeostasis and the transcription factor Zap1 are relevant for cells to thrive under Cu + Fluc conditions. **(A)** The expression of *ZAP1* gene in *Candida glabrata* cells left untreated (Control), treated with 1.25 mM CuSO_4_ (Cu), 32 μg/mL fluconazole (Fluc), or with a combination of Cu and Fluc (Cu + Fluc) was evaluated by qRT-PCR. **(B)** Growth of *Saccharomyces cerevisiae* wild-type (WT) (WT:BY4742) and Δ*zap1* mutant strains in SC agar plates (Control) containing 4 μg/mL of fluconazole (Fluc), 625 μM CuSO_4_ (Cu), or a combination of both (Cu + Fluc). Growth was recorded after 96 h at 30°C. **(C)** Growth of *Candida glabrata* WT and Δ*zap1* mutant strains transformed with a plasmid containing the Cg*ZAP1* gene under the control of its native promoter (<p*ZAP1*>) or with the empty vector (<vector>) in SC agar plates lacking tryptophan (Control) containing 32 μg/mL of fluconazole (Fluc), 625 μM CuSO_4_ (Cu), or a combination of both (Cu + Fluc), after 48 h at 37°C. **(D)** The zinc content of cells untreated (Control) or treated with 625 μM CuSO_4_ (Cu), 32 μg/mL fluconazole (Fluc), or a combination of both was measured by ICP-AES. **(E)** Effect of zinc on the growth sensitivity of *Candida glabrata* cells to Cu + Fluc. Cells were serial diluted and spotted on SD plates containing a mixture of fluconazole (32 μg/mL) and CuSO_4_ (625 μM), supplemented (Cu + Fluc + Zn), or not (Cu + Fluc) with 4 mM of ZnSO_4_. Growth was recorded after 48 h at 37°C. Significance of differences was calculated using student’s *T*-test (**p-value < 0.05; ***p-value < 0.005).

After confirming the transcriptomic data, by qRT-PCR (Figure 8A), we further explored the relevance of Zap1, by evaluating how *ZAP1* deletion affected yeast survival in each individual or combined condition. We tested the knockout effect of the gene in *C. glabrata* and in *S. cerevisiae*, two phylogenetically closely related species (Marcet-Houben and Gabaldón, 2009). Reinforcing the role of Zap1 in yeast survival under these stress conditions, we found that *Sc*Δ*zap1* cells were slightly sensitive to fluconazole and strongly sensitive to copper and Cu + Fluc, with the latter triggering a more pronounced effect (Figure 8B). Mutant *Cg*Δ*zap1* was also sensitive to all stresses, but more prominently to fluconazole and Cu + Fluc (Figure 8C). The tolerance to fluconazole and Cu + Fluc upon reintroduction of Cg*ZAP1* was restored to that of the parent strain (Figure 8C).

We next assessed by ICP-AES whether the downregulation of Cg*ZAP1* (Figure 8D) could impact zinc accumulation. As shown in Figure 8D, the levels of zinc were found to be strongly depleted in *C. glabrata* cells treated with Cu + Fluc but unchanged or even increased under fluconazole and copper conditions, respectively. Zinc supplementation rescued the growth of *C. glabrata* cells treated with Cu + Fluc and increased the brownish pigmentation associated with CuS mineralization (Figure 8E).

Together, these results show that cells exposed to Cu + Fluc fail to uptake zinc and are strongly deprived of this metal, suggesting that Zap1 (and zinc) is important for an adequate response to the stress imposed by the combination of copper and fluconazole.

## Discussion

In recent years, there was a growing interest in exploiting the antifungal properties of copper. Trojan horse strategies that deceive the fungus copper homeostatic mechanisms (Gaspar-Cordeiro et al., 2020; Hunsaker et al., 2020) or the combination of copper with available antifungals (Nagaj et al., 2012; Ząbek et al., 2015; Hunsaker and Franz, 2019b) have proved to be effective against several fungal species. In particular, copper has been shown to potentiate the activity of fluconazole (Ząbek et al., 2015; Hunsaker and Franz, 2019a,b), one of the most prescribed antifungals worldwide. However, despite its interest and great promise, the mechanism underlying this effect remained unknown.

In this work, we have investigated the molecular basis that supports the synergistic effect observed between copper and fluconazole in *C. glabrata* (Figure 1), a yeast intrinsically tolerant to fluconazole and frequently associated with invasive candidiasis (Pfaller et al., 2014, 2019; Whaley and Rogers, 2016).

The analyses of the transcriptome of *C. glabrata* (Figures 3, 4) revealed that the gene encoding a multidrug ABC transporter essential for fluconazole detoxification, *CDR1* (Sanglard et al., 1999), is repressed in response to copper, even in the presence of fluconazole (Figure 5A and Supplementary Tables 2, 4). As copper impairs the activity of Pdr1 (Figure 5D), a Zn_2_Cys_6_ transcription factor known to regulate *CDR1* expression (Vermitsky and Edlind, 2004; Vermitsky et al., 2006), it is possible that *CDR1* repression is a consequence of copper-mediated Pdr1 inhibition. Copper can displace zinc from zinc-finger motifs of transcription factors because of its strong affinity for cysteine-rich domains, which causes structural alterations that lead to the loss of the DNA binding activity (Predki and Sarkar, 1994). Although in this study we have not addressed the effect of copper on Pdr1 binding to DNA, it is clear that the metal also affects the transactivation potential of Pdr1 (Figure 5D), that is, Pdr1’s ability to recruit the basal transcriptional machinery. As a result, in the presence of copper and fluconazole, *C. glabrata* fails to properly activate the fluconazole detoxification response and, therefore, accumulates higher amounts of the drug (Figure 5B).

In agreement with the greater intracellular content of fluconazole, we found that Cu + Fluc treatment elicits a higher accumulation of lanosterol and toxic methylated sterol intermediates (eburicol and 14-α-methylfecosterol). These sterol metabolites alter the physicochemical properties of the plasma membrane, thus affecting membrane-associated processes like transport, growth, and division (Watson et al., 1989; Kelly et al., 1995). Unexpectedly, under co-treatment conditions we found that fluconazole no longer causes ergosterol depletion, as measured by HPLC and confirmed by GC-MS (Figure 6). Although we cannot explain this observation at present, we cannot rule out the possibility of the existence of an as-yet unveiled alternative pathway, specifically activated by copper, which enables the conversion of sterol intermediates into ergosterol, as previously proposed by other authors for other fungi (Huster et al., 2007; Fowler et al., 2011).

Further corroborating the strong deregulation of the sterol metabolism imposed by Cu + Fluc, we noticed that many genes of the ergosterol pathway were downregulated as opposed to fluconazole treatment (Figures 4, 6). In *C. glabrata*, sterol metabolism is controlled by two Zn_2_Cys_6_ transcription factors, Upc2a and Upc2b, orthologs of *S. cerevisiae* Upc2 and Emc22, respectively (Nagi et al., 2011). Upc2a is essential for the activation of *ERG* genes in response to fluconazole treatment, while Upc2b is involved mainly in the uptake of exogenous sterols (Nagi et al., 2011). Studies in *S. cerevisiae* revealed that Upc2 regulates its own expression (Abramova et al., 2001) and that, in response to high levels of ergosterol, it translocates to the cytoplasm (Yang et al., 2015). In this sense, it is possible that under Cu + Fluc treatment, the higher levels of ergosterol (Figure 6) block the activation of Upc2a that would, under normal fluconazole conditions, activate the *ERG* genes (Henry et al., 2000). Another possibility is that copper interferes with the activity of Upc2a *via* zinc-finger disruption. Both options would also explain why the expression of *UPC2A* is downregulated by copper in the presence of fluconazole (Figure 4).

The accumulation of toxic sterol intermediates (Abe et al., 2009), together with the ability of copper to affect membrane permeability and fluidity because of lipid peroxidation and interaction with phospholipids, is highly indicative that membrane alterations might be central for the synergistic effect between copper and fluconazole. Supporting this idea, SEM and TEM analysis revealed pronounced cell surface irregularities, which were more accentuated under Cu + Fluc conditions (Figure 7). As the activity of membrane transporters depends on their correct insertion into the membrane bilayer and their specific interactions with lipids (Lv et al., 2016), it is possible that Cu + Fluc co-treatment compromises metal transport activity, which would explain the decreased accumulation of copper observed in cells treated with Cu + Fluc (Figure 2A). The loss of pigmentation of Cu + Fluc treated cells (Figure 1A) may also be a consequence of impaired function of cell surface-bound enzymes that mineralize CuS to confer resistance to this metal (Yu et al., 1996).

Zap1 is the master regulator of zinc homeostasis in yeasts (Zhao et al., 1998; Nobile et al., 2009), and here we showed that it is important for cells to deal with copper and fluconazole toxicity. The treatment with copper, fluconazole, or a combination of both, represses the expression of *ZAP1*, with such effect being more prominent under copper and Cu + Fluc conditions (Figures 4, 8). Zap1 contains seven zinc-finger motifs in its structure, two of which act as zinc sensors (Bird et al., 2003) and, like Pdr1 (Khakhina et al., 2018), it regulates its own expression (Zhao and Eide, 1997). Therefore, Zap1 activity might as well be impaired by the excess of copper, which would explain the strong *ZAP1* repression under copper and Cu + Fluc conditions (Figure 8A). However, despite a strong downregulation of *ZAP1* by copper, zinc depletion was only evident when cells were simultaneously treated with fluconazole (Cu + Fluc), but not with the metal alone (Figure 8D). We reason that the possible malfunction of membrane metal transporters, due to exacerbated membrane defects observed under Cu + Fluc (Figure 7), may justify this observation. The requirement of zinc for an adequate response to Cu + Fluc is reinforced by the observation that zinc supplementation restores growth under these conditions (Figure 8E). Because zinc is a ubiquitous element, essential for the function of many transcription factors, including Upc2a/b and Pdr1, we propose that, to cope with Cu + Fluc treatment, *C. glabrata* requires adequate zinc levels for these transcription factors to function properly.

A recent study by Hunsaker et al. (2021) evaluated transcriptomic alterations of *C. albicans* cells exposed to a combination of copper and fluconazole. Unlike *C. glabrata*, *C. albicans* cells do not reduce the expression of genes involved in drug detoxification (*CDR1* and *TAC1*), ergosterol metabolism (*ERG* genes), or zinc homeostasis. Also contrasting with our data (Figure 2), in a previous study, the authors showed that co-treatment increases copper accumulation when compared to copper treatment alone (Hunsaker and Franz, 2019a). Therefore, it appears that the mechanisms underlying the synergistic effect between copper and fluconazole are quite distinct between *C. glabrata* and *C. albicans*.

**FIGURE 9:**
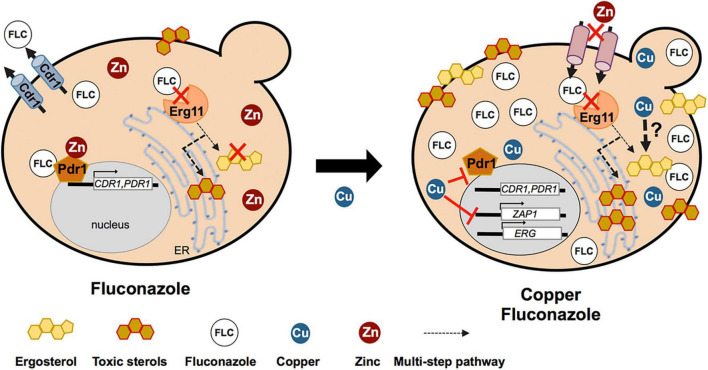
Schematic figure depicting the molecular mechanisms underlying copper and fluconazole synergism. Pdr1 binds to fluconazole (FLC) and activates the expression of genes such as *CDR1*, promoting fluconazole efflux. FLC inhibits Erg11, which leads to blockade of the ergosterol biosynthesis pathway and activation of an alternative sterol pathway that culminate in ergosterol depletion and accumulation of toxic sterols. Copper (Cu) decreases the transactivation activity of Pdr1, rendering cells unable to activate *CDR1* and detoxify FLC. The combination of Cu and FLC also leads to a greater accumulation of toxic sterol intermediates and a consistent downregulation of several genes of the ergosterol pathway (*ERG* genes). However, copper appears to be able to stimulate ergosterol biosynthesis through an as yet unknown mechanism. In addition, Cu and FLC exert a strong inhibition on the expression of *ZAP1*, which causes zinc depletion, further contributing to cellular dysfunction.

In conclusion, our work suggests that copper may negatively impact on the activity of several zinc-finger transcription factors that control important pathways for fluconazole detoxification: azole efflux (Pdr1), ERG (Upc2), and zinc homeostasis (Zap1). Therefore, when copper and fluconazole are combined, cells fail to detoxify the drug and accumulate toxic intermediates of the sterol metabolism. This accumulation, possibly together with copper-induced lipid damages, should affect the activity of membrane metal transporters. Zinc transporters malfunction together with the downregulation of *ZAP1* and its target genes should impede zinc uptake, leading to zinc depletion, which further sensitizes *C. glabrata* cells to copper and fluconazole co-treatment (Figure 9).

Overall, our findings strongly support continued investment in bi-functional drugs, combining azole and copper chelating groups. Such a strategy, while ensuring a synchronized uptake of both compounds, would deceive the yeast copper homeostatic mechanisms, and possibly decrease the effective copper concentration needed to achieve the same synergistic effect, which would certainly reduce the toxicity to human cells.

## Data Availability Statement

The datasets presented in this study can be found in online repositories. The names of the repository/repositories and accession number(s) can be found below: https://www.ncbi.nlm.nih.gov/geo/, GSE162741.

## Author Contributions

AG-C and CP contributed to the conceptualization and wrote the original draft. AG-C, CA, WA, and AM performed the experiments. AG-C, CA, CP, VP, and AP analyzed the data. PP performed the clinical sample collection. CP performed the project administration and funding acquisition. All authors contributed to writing – review and editing of the manuscript.

## Conflict of Interest

The authors declare that the research was conducted in the absence of any commercial or financial relationships that could be construed as a potential conflict of interest.

## Publisher’s Note

All claims expressed in this article are solely those of the authors and do not necessarily represent those of their affiliated organizations, or those of the publisher, the editors and the reviewers. Any product that may be evaluated in this article, or claim that may be made by its manufacturer, is not guaranteed or endorsed by the publisher.
