# Supplementary material for: Copper Acts Synergistically With Fluconazole in Candida glabrata by Compromising Drug Efflux, Sterol Metabolism, and Zinc Homeostasis
Source: Front Microbiol. 2022 Jun 14;13:920574. doi: 10.3389/fmicb.2022.920574 (PMC9237516; doi:10.3389/fmicb.2022.920574)
Supplement: Supplementary file 1 [file Data_Sheet_1.PDF]

Copper Acts Synergistically with Fluconazole in *Candida glabrata* by Compromising Drug Efflux, Sterol Metabolism, and Zinc Homeostasis

Running title: Copper and fluconazole synergy in *Candida glabrata*

[1]Gaspar-Cordeiro, A., [1]Amaral, C., [1]Pobre, V., [1,2]Antunes, W., [1]Petronilho, A. [3]Paixão, P., [4]Alves de Matos, A.P., C., [1*]Pimentel, C.

[1] Instituto de Tecnologia Química e Biológica António Xavier, Universidade Nova de Lisboa, Av. República, 2780-157 Oeiras, Portugal.
[2] Centro de Investigação da Academia Militar (CINAMIL), Unidade Militar Laboratorial de Defesa Biológica e Química (UMLDBQ), Av. Dr. Alfredo Bensaúde, 1849-012 Lisboa, Portugal.
[3] Unidade de Infeção. Chronic Diseases Research Centre - CEDOC. NOVA Medical School/Faculdade de Ciências Médicas. Universidade NOVA de Lisboa. Lisboa; Laboratório de Patologia Clínica - SYNLAB. Hospital da Luz. Lisboa. Portugal.
[4] Egas Moniz Interdisciplinary Research Centre, Egas Moniz Higher Education Cooperative, Caparica, Portugal.
*corresponding author: pimentel@itqb.unl.pt

Supporting Information Content:

- Table S1 – Strains used in this study.
- Table S2 - Genes differentially expressed in *Candida glabrata* cells treated with copper (submitted as .xslx)
- Table S3 - Genes differentially expressed in *Candida glabrata* cells treated with fluconazole (submitted as .xslx).
- Table S4 - Genes differentially expressed in *Candida glabrata* cells treated with copper and fluconazole (submitted as .xslx).
- Table S5 - Genes differentially expressed in *Candida glabrata* cells treated with copper and fluconazole in comparison to fluconazole alone (submitted as .xslx).
- Figure S1
- Figure S2

Table S1– Strains used in this study.

Strain	Genotype/ Description	Source
C.glabrata ATCC2001	Wild-type strain	ATCC
C. glabrata HTL	his3Δ::FRT leu2Δ::FRT trp1Δ::FRT	(Schwarzmüller et al., 2014)
C. glabrata HTU	his3Δ::FRT ura3Δ::FRT trp1Δ::FRT	(Jacobsen et al., 2010)
C. glabrata BVGC3	ATCC2001 ERG11-3× HA	(Vu et al., 2019)
C. glabrata *Δcdr1*	his3Δ::FRT leu2Δ::FRT trp1Δ::FRT CAGL0M01760g::NAT1	(Schwarzmüller et al., 2014)
C. glabrata *Δzap1*	leu2Δ::FRT trp1Δ::FRT CAGL0J05060g::his3Δ	This study
S. cerevisiae BY4742	MATα his3Δ1 leu2Δ0 lys2Δ0 uraΔ0	Euroscarf
S. cerevisiae *Δzap1*	MATα his3Δ1 leu2Δ0 lys2Δ0 uraΔ0 YJL056c::kanMX4	Euroscarf
H17	*Candida glabrata* clinical strain	vaginal mucosa

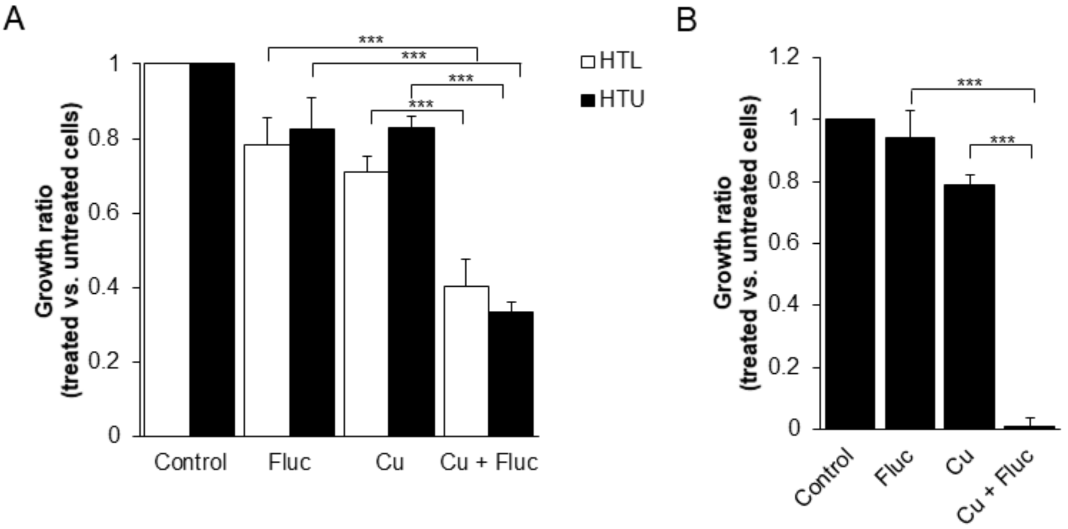

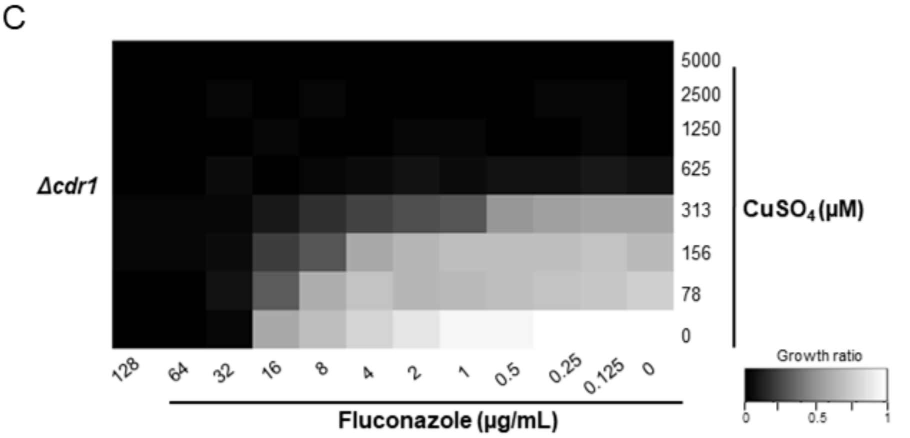

ΣFIC								CuSO₄ (μM)
0.16	0.28	0.53	0.53	0.53	1.03	1.03	1.03	**313**
0.38	0.5	0.75	0.75	0.75	1.25	1.25	1.25	**156**
0.63	0.75	1	1	1	1.5	1.5	1.5	**78**
**16**	**8**	**4**	**2**	**1**	**0.5**	**0.25**	**0.125**	

Fluconazole (μg/mL)

ΣFIC ≤ 0.5 – synergy

D

**Strain**	**MIC$_{flu}$ (μg/mL)** Broth microdilution assay MIC$_{50}$	MIC$_{90}$
H17	64-32	>128
HTU	64-32	64-128
HTL	64-32	64-128
Δcdr1	32-16	32-16

**Strain**	**IC$_{Cu}$ (μM)** Broth microdilution assay IC$_{50}$	IC$_{90}$
H17	1.25-0.63	1.25-0.63
HTU	0.31-0.16	1.25 -0.63
HTL	0.63-0.31	1.25 -0.63
Δcdr1	0.63-0.31	0.63-0.31

Figure S1 – Effect of the combination of copper and fluconazole on the growth of A: different laboratory (HTL and HTU) and B: H17 clinical strain of *Candida glabrata*. Cultures

were incubated for 24 h at 37 ºC in the presence of 156 µM (laboratory) or 313 µM (clinical) of $CuSO_4$ (Cu), 32 µg/mL fluconazole (Fluc) or both (Cu+Fluc), and growth was compared to that of untreated cultures (Control). C: Checkerboard assays. *CgΔcdr1* cells were exposed to all the possible combinations of $CuSO_4$ and fluconazole (ranging between 5000 – 78 µM for $CuSO_4$ and 128 – 0.125 µg/mL for fluconazole). Growth ratios were evaluated after 24 h at 37 ºC by measuring OD_{600} and normalizing it relative to untreated controls (see gradient bar). The Fractional Inhibitory Concentration (FIC) index (ΣFIC) was calculated for the indicated combinations of fluconazole and $CuSO_4$. The dashed red squares indicated the Cu+Fluc concentrations where synergy is observed. D: Tables indicating the MIC of fluconazole (MIC_{flu}) and the IC of $CuSO_4$ (IC_{Cu}) for all the strains used in this figure. MICs and ICs were evaluated after 24 h at 37 ºC with the respective compounds.

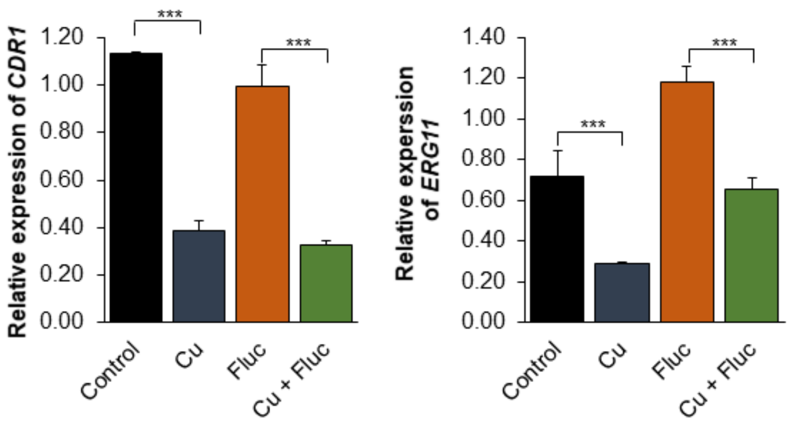

Figure S2 – Relative expression of *CDR1* and *ERG11* genes in *C. glabrata* cells exposed to 156 µM $CuSO_4$ (Cu), 16 µg/mL fluconazole (Fluc) or both compounds (Cu+Fluc) for 2 h. Significance of differences was calculated using a student's T-test (*** p-value < 0.005).

References:

Jacobsen I D, Brunke S, Seider K, Schwarzmüller T, Firon A, D'enfért C, Kuchler K, Hube B (2010). Candida glabrata persistence in mice does not depend on host immunosuppression and is unaffected by fungal amino acid auxotrophy. Infect Immun, 78(3): 1066-1077

Schwarzmüller T, Ma B, Hiller E, Istel F, Tscherner M, Brunke S, Ames L, Firon A, Green B, Cabral V, Marcet-Houben M, Jacobsen I D, Quintin J, Seider K, Frohner I, Glaser W, Jungwirth H, Bachellier-Bassi S, Chauvel M, Zeidler U, Ferrandon D, Gabaldón T, Hube B, D'enfert C, Rupp S, Cormack B, Haynes K, Kuchler K (2014). Systematic phenotyping of a large-scale Candida glabrata deletion collection reveals novel antifungal tolerance genes. PLoS Pathog, 10(6): e1004211

Vu B G, Thomas G H, Moye-Rowley W S (2019). Evidence that Ergosterol Biosynthesis Modulates Activity of the Pdr1 Transcription Factor in Candida glabrata. mBio, 10(3)
